# Machine learning and artificial intelligence: applications in healthcare epidemiology

**DOI:** 10.1017/ash.2021.192

**Published:** 2021-10-07

**Authors:** Alisa J. Hamilton, Alexandra T. Strauss, Diego A. Martinez, Jeremiah S. Hinson, Scott Levin, Gary Lin, Eili Y. Klein

**Affiliations:** 1 Center for Disease Dynamics, Economics & Policy, Silver Spring, Maryland, United States; 2 Department of Medicine, Johns Hopkins University, Baltimore, Maryland, United States; 3 School of Industrial Engineering, Pontificia Universidad Católica de Valparaíso, Valparaíso, Chile; 4 Department of Emergency Medicine, Johns Hopkins University, Baltimore, Maryland, United States

## Abstract

Artificial intelligence (AI) refers to the performance of tasks by machines ordinarily associated with human intelligence. Machine learning (ML) is a subtype of AI; it refers to the ability of computers to draw conclusions (ie, learn) from data without being directly programmed. ML builds from traditional statistical methods and has drawn significant interest in healthcare epidemiology due to its potential for improving disease prediction and patient care. This review provides an overview of ML in healthcare epidemiology and practical examples of ML tools used to support healthcare decision making at 4 stages of hospital-based care: triage, diagnosis, treatment, and discharge. Examples include model-building efforts to assist emergency department triage, predicting time before septic shock onset, detecting community-acquired pneumonia, and classifying COVID-19 disposition risk level. Increasing availability and quality of electronic health record (EHR) data as well as computing power provides opportunities for ML to increase patient safety, improve the efficiency of clinical management, and reduce healthcare costs.

Attempts to harness the power of computing to generate “artificial intelligence” began with Alan Turing in the 1940s. During and after World War II, Turing developed theories about what constituted artificial intelligence (AI) that still resonate today (eg, the Turing test), and he wrote about how to create computers that “can learn from experience.”^1^ At the time, AI remained largely theoretical due to limitations in computing power. Today, AI is widely used to augment diverse areas of human experience, including internet searches, robotics, policing, and disease diagnosis and treatment. Although the definition of AI is broad and has evolved over the years, AI generally refers to the performance of tasks by machines ordinarily associated with human intelligence. Machine learning (ML) is a subtype of AI that refers to the ability of computers to draw conclusions (ie, learn) from data without being programmed directly.^2,12^

Machine learning builds from traditional statistical methods and has drawn significant interest in healthcare epidemiology due to its potential for improving disease prediction and patient care. Advantages include its ability to leverage large-scale, highly dimensional data from electronic health record (EHR) systems, to conduct variable selection as part of model building, and to identify interactions in data to subgroup patients with respect to outcomes.^3–8^ In this review, we summarize ML in healthcare epidemiology and provide practical examples of ML tools used to support decision making at 4 stages of hospital-based care: triage, diagnosis, treatment, and discharge. Relevant ML terms are summarized in Table 1.

**Table 1.** Relevant Machine Learning Terms

Term	Definition
Artificial intelligence (AI)	A computer’s ability to learn from experience
Machine learning (ML)	A type of artificial intelligence in which computers draw conclusions from data without being directly programmed
Supervised learning	Models in which the outcome is known for each observation
Unsupervised learning	Models in which the outcome is not known for each observation
Semisupervised learning	Models in which the outcome is known for some observations but not others
Label	The patient outcome (dependent variable)
Feature	An attribute/characteristic of the patient (dependent variable)
Sensitivity	Ability of a model to correctly identify true cases
Specificity	Ability of a model to directly identify negative cases
Accuracy	Measure of correctly labeled data instances over the total number of instances
Precision	Fraction of relevant instances among the retrieved instances (ie, positive predictive value)
Recall	Fraction of relevant instances that were retrieved correctly (ie, sensitivity)
Training data set	Data used to develop a model
Validation data set	Data used to test a model’s performance while training
Test data set	Data used to test the accuracy, precision, or recall against real-world data
Out-of-sample data	In a study cohort, the data not used as training data
Bias-variance tradeoff	In supervised learning, overfitting and underfitting can result in loss of performance
Bias	Difference between the average prediction of a model and the correct value
Variance	Variability of a model prediction for a given data point
Overfitting	When the model follows noise, resulting in low bias and high variance
Noise	Nonpredictive features in the data set
Underfitting	When the model fails to capture the underlying patterns in the data, resulting in low variance and high bias
Decision tree	A model that separates data into smaller and smaller partitions until each observation is classified according to the outcome of interest
Stopping criteria	Criteria used to stop further partitioning of data in a decision tree. Can prevent overfitting
Ensemble model	An ML technique combining multiple individual models
Random forest	A type of ensemble model that combines decision trees to produce a probabilistic prediction for the outcome
Receiver operator characteristic (ROC) curve	A way to graph the sensitivity and specificity (or precision) of a model
Area under the curve (AUC)	A technique to compare model results (with other models or other measurement tools) by calculating the area under an ROC curve
Natural language processing (NLP)	A type of AI in which the algorithm learns how to ‘understand’ language, including contextual nuances

## Types of learning and algorithms

Machine-learning algorithms can identify relationships between patient attributes and outcomes to construct models that can make predictions for new and unseen patients and can group patients based on similar attributes.^9^ Although there is overlap, the simplified difference between statistical methods and ML is that statistics is generally associated with drawing inferences from data, whereas ML is more concerned with finding generalizable predictive patterns.^10^ Thus, while statistics uses algorithms to learn about a model’s attributes from the data assuming the model’s structure, ML harnesses computing power and uses algorithms to learn about the model’s structure and attributes directly. Although statistics and ML are both concerned with how we learn from data, ML methods focus largely on prediction as opposed to explanation or causal inference.^11^

Machine-learning algorithms utilize 3 methods of ‘learning’: supervised, unsupervised, and semisupervised.^12^ In supervised learning, the outcome (ie, the dependent variable or ‘label’) is known for each patient. Fed structured data for patient attributes (ie, the independent variables or ‘features’), the algorithm attempts to find the corresponding model that predicts patient outcomes with the highest precision, accuracy, or recall. In unsupervised learning, the algorithm attempts to establish relationships between patient features without knowing outcomes to group patients based on their similarities. In semisupervised learning, the model is fit to both labeled and unlabeled data; this can be useful for large data sets for which labeling data is very time consuming.^12^

Box 1: Bias-Variance Tradeoff

Common algorithms used in ML include decision trees, random forest, naïve Bayes, k-means clustering, and ensemble models (combinations of individual models). Traditional statistical methods, such as generalized linear models and Cox proportional hazards, can also be adapted using ML to make predictions. Each has their advantages and disadvantages, but all are subjected to the bias-variance tradeoff referring to a model’s tendency to either overfit or underfit the data and resulting in a loss of performance.^13^ Overfitting occurs when the model follows noise (or irrelevant features in the data set), resulting in low bias and high variance. Underfitting occurs when a model is unable to follow the patterns in the data set correctly, resulting in high bias and low variance. The goal is to optimize the model so that both bias and variance are reduced.

Applications of ML in healthcare epidemiology have tended to rely on supervised learning. The pipeline of ML tool development can be simplified into 4 steps. (1) Researchers assemble a retrospective data set of routinely collected EHR data (eg, age, gender, vital signs, comorbidities, or emergency department (ED) presentation). (2) A subset of this data is used as input data or training data and fit to 1 or more algorithms, allowing the computer to learn how different patient features interact to predict each patient’s outcome. (3) The resulting model is then evaluated on a validation data set, and the model with the highest accuracy, precision, and recall is selected. Often the validation data set is created from the subset of cohort data not used as training data and referred to as out-of-sample data. (4) After the training process, a test data set is used to compare the predicted outcomes of the selected model to real-world patient outcomes. If the model performs well, it can be used prospectively in combination with clinician expertise to inform treatment decisions. To assess model performance in the real world, it is common for models to be run in the background and to record their predictions without presenting them to providers until results are accurate and unlikely to adversely impact patients. The use of ML in healthcare is already common, and ML can be utilized across the continuum of care. Here we present examples of some ways that ML has been used to improve decision making through the course of a hospitalization.

## Triage: Emergency medicine

The first encounter with the hospital for many patients is the emergency department, where patients are triaged by acuity level to prioritize care for the most severely ill patients. However, overcrowding is a major problem in emergency medicine. Demand for care that exceeds supply drives long wait times and delays that have been strongly associated with worse health outcomes.^14^ Most EDs in the United States use the rule-based, 5-stage Emergency Severity Index (ESI),^15^ which relies largely on provider judgment to assign incoming patients to triage acuity levels. ESI level 1 denotes highest acuity (ie, the patient needs immediate treatment) whereas level 5 denotes lowest acuity (ie, treatment needs are nonurgent). Clinicians must rapidly assess patients with diverse medical conditions using limited information and quickly decide whether a patient needs immediate care or can safely wait.^3^ Using standard tools, such as the ESI, triage acuity designations are highly variable between providers and not well-correlated with risk of adverse outcome.^16,17^ Additionally, more than half of patients in the United States are assigned to ESI level 3,^18,19^ a middle-tier risk designation that is associated with prolonged waiting.

To address these issues, Levin et al^3^ used ML to develop an ED triage system (‘e-triage’) to assist clinicians in performing more accurate and consistent triage and to distribute patients across risk designations to optimize operations and facilitate rapid care delivery. The sample included a retrospective cohort of 172,726 adult visits to an urban and community ED. Researchers generated random forest models to predict 3 outcomes in parallel: (1) critical care (ie, in-hospital mortality or direct admission to the intensive care unit, ICU), (2) emergency procedure (ie, any surgical procedure within 12 hours of arrival), and (3) hospitalization (ie, admission to an inpatient care site or transfer to an external acute care hospital). Outcome probabilities were then mapped to 1 of 5 e-triage acuity levels, similar to ESI. For example, patients with >15% likelihood of needing critical care or an emergency procedure were assigned to e-triage level 1. Accuracy of e-triage predictions was measured using out-of-sample area under the receiver operator characteristic curve (AUC) and compared to actual patient ESI levels. Measures of difference were reported as ‘equivalent,’ ‘up-triage’ (ie, e-triage predicted a higher risk than ESI), or ‘down-triage’ (ie, e-triage predicted a lower risk than ESI). Compared to manual triage, those who would have been up-triaged by e-triage were 5 times more likely to experience the critical care or emergency surgery outcome and twice as likely to be hospitalized. Those down-triaged had a lower likelihood of these outcomes. The model was implemented as an aid to decision makers (not as the final arbiter of triage designation), which increased acceptance and resulted in improved resource allocation and reduction in wait times for patients.

BOX 2: Random Forest and Area Under the Receiver Operator Characteristic Curve

Random forest models combine multiple decision trees. A decision tree is a ML model, making random forest a type of ensemble model. A decision tree starts with a question about the independent variables of an observation then assigns a binary classification based on the answer.^12^ All observations move down the branches of the tree until the stopping criteria are reached and outcomes determined. A random forest model trains a set of decision trees and aggregates output to produce a probabilistic prediction for each outcome.^3^ A receiver operator characteristic (ROC) curve is a common way to graph the results of a model or measurement tool. The y-axis most often represents the true-positive rate (sensitivity). The x-axis usually represents the false-positive rate (1-specificity) but may also represent precision or the proportion of true cases correctly classified. The curve is created by plotting points corresponding to all probability thresholds between 0 and 1, and the model or measurement tool with the largest area under the curve (AUC) is considered the most effective.

## Diagnosis: Septic shock

Throughout a patient’s stay in the hospital, numerous decisions are made, and diagnoses may be missed. In particular, septic shock, which is responsible for 10% of ICU admissions, 20%–30% of hospital deaths, and $15.4 billion in annual healthcare costs,^20–23^ is of critical importance. Research shows that early detection and treatment of septic shock reduces morbidity, mortality, and length of stay.^20,23–26^ A growing body of research has explored the utility of ML to predict septic shock based on data from bedside monitors^27,28^ and routine measurements for septic shock prediction.^29–31^ Henry et al^20^ were the first to use ML and EHR data to develop a scoring system (ie, ‘TREWscore’) that predicts septic shock hours before onset.

Using supervised learning, researchers fit a Cox proportional hazards model^32,33^ to identify a subset of features most indicative of septic shock and generated a risk prediction score over time. Input features (predictors) included physiological markers (eg, heart rate, respiratory rate, and white blood cell count) as well as derived measures based on expert opinion (eg, systemic inflammatory response syndrome (SIRS) criteria^34^). Risk scores were compared to actual patient outcomes and to 2 existing screening tools: (1) MEWS, a severity score for ICU triage in surgical patients also used for sepsis screening^35^ and (2) a routine septic shock screening protocol that identifies patients with suspicion of infection and either hypotension or hyperlactatemia.^36,37^ The predicted risk score had a higher sensitivity than both MEWS and the routine screening tool, and it correctly identified septic patients a median of 28.2 hours before septic shock onset and 7.43 hours before sepsis-related organ dysfunction. Implemented throughout the hospital system, it routinely alerts clinicians to the possibility of sepsis allowing earlier intervention.

## Treatment: Community-acquired pneumonia

Clinicans routinely initiate empirical antibiotic therapy while waiting for laboratory results. This is particularly true for possible upper respiratory infections, including community-acquired pneumonia (CAP), which is difficult to diagnose. In the United States, there are an estimated 4–6 million annual cases of CAP, and CAP is responsible for 600,000 to 1.1 million hospitalizations and >$17 billion in health expenditures each year.^38–42^ CAP is a major driver of hospital antibiotic use, which contributes to antibiotic resistance. CAP can be difficult to identify, and treatment is often suboptimal due to incorrect choice of therapy, dose, route, or duration. Patients may also be prescribed treatment when they do not actually have CAP. Rapid correction of inappropriate therapy can improve patient outcomes and reduce the risk of antibiotic resistance. To address this problem, Fabre et al^43^ used ML models to prospectively identify CAP patients.

Model building used a similar approach to previous examples. The first step, however, was identifying patients who actually had CAP and those who did not. Because no discrete mechanism for identifying CAP patients has been developed, researchers manually identified patients through chart review. Initial models utilized physiological markers (eg, vital signs and laboratory data) in EHR data captured through routine clinical care. However, predictions were hampered by a lack of highly predictive discrete elements. To improve model predictions, researchers used another type of AI called natural language processing (NLP)^12,44^ to establish relationships between free-text notes by clinicians and the outcome.

NLP refers to algorithms capable of ‘understanding’ the contents of a document, including textual nuances, such as negation statements (eg, the patients does not have pneumonia). This type of technology underlies common customer service chat bots, spell check applications, Google translate, and digital assistants. Free-text indicators in the CAP model included chief complaint of fever or chills, radiographic report of consolidation, and radiographic report of infiltrate. Inclusion of the NLP-derived variable ‘consolidation’ dramatically improved the model’s ability to predict CAP patients, exemplifying how the application of ML strategies can address the challenge of syndrome-based antibiotic stewardship.

## Discharge: COVID-19 disposition

Finally, when patients leave the hospital, several decisions need to be made about care. This has been particularly true during the COVID-19 pandemic, which has put immense strain on healthcare systems across the United States. Many hospitals have been overrun with patients and have been forced to create new patient management systems to optimize allocation of limited space. Prediction of clinical trajectory in patients with this novel and sometimes critical disease is difficult and was a major challenge to disposition decision making early in the pandemic. Emergency department clinicians have been tasked with determining which patients most need admission to hospital wards or ICUs, which patients can be transferred to field hospitals, and which patients can be discharged home. These decisions have often been made very early in the disease course and with limited information. To address this challenge, Hinson et al developed a ML algorithm to predict near-term clinical deterioration in ED patients under investigation for COVID-19, and paired model-generated outcome probabilities with EHR-integrated disposition decision support (unpublished data).

Utilizing real-time EHR data, including ED chief complaint, active medical problems, vital signs, oxygen support, and laboratory results, researchers used a random forest model to generate probabilistic risk estimates for 2 composite outcomes: (1) cardiopulmonary failure within 24 hours and (2) cardiopulmonary dysfunction within 72 hours from discharge. Cardiopulmonary failure was defined as death, respiratory failure requiring high-volume oxygen or mechanical support, or cardiovascular failure requiring vasopressors or admission to the intermediate care unit (IMC) or ICU. Cardiovascular dysfunction was defined as at least moderate organ dysfunction that required hospital-based interventions (eg, oxygen administration, intravenous fluid administration). Risk threshold determination was used to map outcome probabilities to 1 of 10 COVID-19 clinical deterioration risk levels, with level 10 being most severe.

This tool was rapidly implemented in the clinical environment and was used to support care decisions within the Johns Hopkins Health System during the pandemic. To support decision making in real time, risk levels were presented in the EHR alongside a continuum of dispositions to be considered by providers, including admission to IMC/ICU, admission to a ward, transfer to a field hospital, or discharge. The tool drove more consistent and reliable disposition decision making and improved bed allocation across the health system.

## Discussion

As demonstrated in the examples presented, the real-world applications of ML can optimize patient care throughout several stages of hospitalization. ML prediction models are not meant to replace provider judgment, but they can be used as a tool to assist decision making and to help clinicians identify potential treatment pathways. The increasing availability of EHR data and other sources provides ML opportunities to learn more about disease prevention, classification, and trajectory and to develop earlier and more targeted interventions.^44^ Models may not be 100% accurate but when supplemented with clinical expertise, they can be helpful and can improve health outcomes.^45^

Machine learning can also be helpful outside risk prediction, for example, in designing more efficient clinical trials and generating testable hypotheses.^44^ Clinical trials investigating rare diseases may be underpowered because too small a proportion of the study population has the outcome. ML can be used to identify patients with the disease and to generate a large enough intervention group for an adequately powered study with fewer participants.^44^ ML models are helpful to predict which factors lead to increased risk but do not explain exactly why or how. Narrowing down predictive factors can inform hypotheses in investigations of the biological and behavioral mechanisms behind disease trajectories and transmission.^44^

Machine learning models work best with large amounts of high-quality data, and their utility is limited by data inconsistencies, inaccuracies, and errors.^44^ Furthermore, a model can only identify relationships that present in the data.^44^ Some models, such as decision trees, may be prone to overfitting; they work well with training data or at a certain institution but poorly with new data or in a different context (ie, they are not generalizable).

Selection bias due to missing data or oversampling in healthcare and public health is a challenge that exacerbates health disparities.^46^ Models developed from unrepresentative data will produce biased predictions. For example, an algorithm designed to visually recognize skin cancer will worsen racial disparities in dermatology if it is not tested on data from people of color.^47^ Additionally, vulnerable populations without adequate access to healthcare will be underrepresented in EHR systems. Some efforts have been made to assess the extent of missing clinical data.^48^ ML has also been used to identify when standard scoring systems accentuate racial disparities, and models have been designed with the aim of reducing racial bias in outcome predictions.^49^

In addition to bias and computational challenges, ML projects introduce the same challenges of any interdisciplinary research project aiming to inform practice and policy. Developing a practical model requires expertise from healthcare epidemiologists, clinicians, computer scientists, and other professionals. Results and application then need to be communicated to public health officials, hospital administrators, and researchers. Currently, a standardized approach to model building in healthcare epidemiology has not yet been established, which can lead to a lack of transparency and hamper reproducibility. A lack of transparency is further compounded with complex ‘black box’ models, in which the reasons behind risk-factor selection are obscured.

Although ML algorithms can be highly predictive, models contribute little to patient outcomes without adoption by providers.^50–52^ Often overlooked in development, implementing ML models as clinical decision support tools often faces significant challenges due to system factors such as lack of computational resources or regulatory requirements that limit data sharing. Another challenge is determining where to present model results to providers within the care model. For example, a decision support tool needs to present recommendations at the point of decision and provide alternatives, not just state that certain choices may be incorrect. Interface design is also important to consider; electronic interfaces that are not user friendly or that rely on computer literacy and user skill may illicit resistance from providers.^53^ Implementation of alerts, such as the sepsis alert described above, have 2 implementation issues: (1) they need to be specific enough to avoid alert fatigue and (2) they need to be implemented in a way that does not disrupt provider work flow.^53,54^

To date, ML has proven to be a helpful tool in increasing patient safety, improving the efficiency of clinical management, and reducing healthcare costs.^53^ Successful efforts to implement ML algorithms, like the ones highlighted in this article, will increase support for efforts to improve data collection and promote consistency and clarity across EHR systems and user interfaces and standardization in model building. Such efforts will ultimately lead to more accurate models, valuable clinical decision support, and better health outcomes. Continued increases in computing power and advances in ML will likely lead to improved predictive power and increased efforts to embed algorithms into clinical care. Although ML models can be useful, they need to be implemented in a manner that can augment clinician decision making. As with all advances in computation in medicine, we must proceed with caution and care, including both clinicians and patients in the process, to ensure that models actually improve patient outcomes.
